# Perioperative mortality among trauma patients in Northwest Ethiopia: a prospective cohort study

**DOI:** 10.1038/s41598-023-50101-8

**Published:** 2023-12-21

**Authors:** Amanuel Sisay Endeshaw, Eshetu Tesfaye Dejen, Bekalu Wubshet Zewdie, Biniyam Teshome Addisu, Misganew Terefe Molla, Fantahun Tarekegn Kumie

**Affiliations:** 1 Department of Anesthesia, College of Medicine and Health Science, Bahir Dar University, Bahir Dar, Ethiopia; 2 Department of Orthopedics and Traumatology, College of Medicine and Health Science, Bahir Dar University, Bahir Dar, Ethiopia

**Keywords:** Outcomes research, Trauma

## Abstract

Trauma is the leading cause of mortality in persons under 45 and a significant public health issue. Trauma is the most frequent cause of perioperative mortality among all surgical patients. Little is known about perioperative outcomes among trauma patients in low-income countries. This study aimed to assess the incidence and identify predictors of perioperative mortality among adult trauma victims at Tibebe Ghion Specialised Hospital. From June 1, 2019, to June 30, 2021, a prospective cohort study was conducted at Tibebe Ghion Specialized Hospital. Demographic, pre-hospital and perioperative clinical data were collected using an electronic data collection tool, Research Electronic Data Capture (REDCap). Cox proportional hazard model regression was used to assess the association between predictors and perioperative mortality among trauma victims. Crude and adjusted hazard ratio (HR) with a 95% confidence interval (CI) was computed; a p-value < 0.05 was a cutoff value to declare statistical significance. One thousand sixty-nine trauma patients were enrolled in this study. The overall incidence of perioperative mortality among trauma patients was 5.89%, with an incidence rate of 2.23 (95% CI 1.74 to 2.86) deaths per 1000 person-day observation. Age ≥ 65 years (AHR = 2.51, 95% CI: 1.04, 6.08), patients sustained blunt trauma (AHR = 3.28, 95% CI: 1.30, 8.29) and MVA (AHR = 2.96, 95% CI: 1.18, 7.43), trauma occurred at night time (AHR = 2.29, 95% CI: 1.15, 4.56), ASA physical status ≥ III (AHR = 3.84, 95% CI: 1.88, 7.82), and blood transfusion (AHR = 2.01, 95% CI: 1.08, 3.74) were identified as a significant predictor for perioperative mortality among trauma patients. In this trauma cohort, it was demonstrated that perioperative mortality is a healthcare burden. Risk factors for perioperative mortality among trauma patients were old age, patients sustaining blunt trauma and motor vehicle accidents, injuries at night, higher ASA physical status, and blood transfusion. Trauma care services need improvement in pre-hospital and perioperative care.

## Introduction

Every year globally, 5.8 million people die annually from injuries resulting from trauma, making it the leading cause of death among people under 45 and a significant public health challenge^1^. According to projections by the World Health Organization (WHO), there is expected to be a 40% rise in fatalities resulting from trauma by 2030. Of all trauma-related deaths worldwide, about 90% occur in low- and middle-income countries (LMICs)^2^. Apart from mortality, millions of disability-adjusted life years (DALYs) are lost due to injuries, with Sub-Saharan Africa earing the largest burden^3^.

Recent improvements in trauma care in pre-hospital care, emergency department care, operation techniques, and intensive care led to a constant reduction of mortality rates in high-income countries, but the problem is still not addressed in LMIC^4^. Additionally, in developed countries, late deaths following traumatic injuries have been significantly reduced due to advancements in trauma care, resulting in a shift of mortality patterns from trimodal to bimodal^5,6^. However, in underdeveloped countries, time to death following trauma still follows the traditional trimodal pattern in which there have been first, second, and third peaks of death in which deaths occur in the first minute, within 4 h, and weeks, respectively^7^.

Surgical interventions are the principal treatment delivered to trauma patients to reduce mortality, especially in the 3rd peak of death following traumatic injuries. Studies from developing countries reported that nearly one-fourth of trauma patients died within 30 days of surgery^8^. Furthermore, trauma is a significant cause of perioperative mortality in all surgical patients^9^. Despite limited information about risk factors for perioperative mortality in LIC, delayed surgery is one of the most significant risk factors for perioperative mortality among trauma patients in low-income countries^10^. Other risk factors for perioperative mortality among trauma patients in LIC include age, severity of injury, and comorbidities^11^.

Among sub-Saharan countries, Ethiopia has one of the highest road traffic injury-related mortality^12^. it is double burdened by a poor trauma care system and inadequate surgical facilities^13,14^. However, there is scarce information about perioperative outcomes among African trauma patients. Therefore, this study aimed to assess the magnitude and risk factors associated with perioperative mortality among trauma patients in Ethiopia.

## Methods

### Study design and period

From June 1, 2019, to June 30, 2021, a prospective cohort study was conducted at Tibebe Ghion Specialized Hospital. We used the Strengthening the Reporting of Observational Studies in Epidemiology (STROBE) checklist for reporting this study.

### Clinical setting

This study was conducted at Tibebe Ghion Specialized Hospital, a tertiary-level hospital located ta Bahir Dar City, Northwest Ethiopia. Both teaching and clinical services are affiliated with Bahir Dar University, College of Medicine and Health Science. The hospital provides a 24/7 trauma and emergency service by emergency medical doctors and nurses. With various specialist and subspecialist medical doctors, trauma and orthopedic, general, head and neck, maxillofacial, pediatric, cardiothoracic, gynecologic and obstetrics, urologic, and ophthalmic surgeries will be provided accordingly for trauma victims. In addition, Basic and advanced radiologic services such as X-ray, ultrasound, CT-scan, and MRI are available for trauma victims.

The hospital has 11 major and two minor operation rooms for all elective and emergency surgery.

### Eligibility criteria

All adults aged ≥ 18 trauma victims who underwent surgery were eligible for this study. We excluded trauma victims with no cell phone and family members for follow-up.

### Study variables

The dependent variable for this study was time to death in days until the 28th day after surgery. The explanatory variables were (1) demographic: age, sex, (2) pre-hospital variables: time to hospital arrival, mode of transport, injury mechanism, the time frame of trauma, (3) perioperative: ASA physical status, comorbidity, the urgency of surgery, anesthesia type, procedure, hemoglobin, preoperative systolic blood pressure (SBP), blood loss, blood transfusion.

### Operational definitions

#### Event

Trauma victims who died within 28 days following surgery.

#### Censored

Trauma victims who were alive at 28 days following surgery.

#### Trauma patient

A patient sustained a tissue injury that occurs more or less suddenly due to violence or accident and is accountable for initiating hypothalamic–pituitary–adrenal axis, immunologic and metabolic responses that are responsible for restoring homeostasis^15^.

#### Elective surgery

Trauma patients who are operated on an elective base after the stabilization of acute conditions^16^.

### Data collection procedure and quality control

A previously reported data collection tool for prospectively collecting perioperative data for surgical cases in LMIC was used^17^. An internet based system with the option of offline data entry data collection system called Research Electronic Data Capture (REDCap) was used. Using electronic tablets, the anesthetist or anesthesia student at Tibebe Ghion Specialised Hospital completes the data collection tool's initial demographic, preoperative vital signs, and surgical and anesthetic information sections. After receiving initial information, one dedicated data manager follows the patient and finishes the follow-up data, including the mortality status.

#### Follow-up

Until discharge, patients were followed in person; then weekly follow-up phone calls up to 28th postoperative days. Multiple phone numbers were obtained from family members to reduce dropout.

The data manager routinely verified the collected data with the hospital's logbook. Overall, the data collection system, including the electronic tables functionality, was checked consistently by one information technology person as part of the research team. Simulated training regarding the data collection system was given to all involved in the data collection. Finally, the collected data were stored in a secured REDCap database.

### Data analysis

Data were analyzed and reported using R software version 4.1.4 (R Foundation for Statistical Computing, Vienna, Austria). "survival" package was used to conduct survival analysis in R. Descriptive statistics were used to show the characteristics of the study subjects using tables and graphs. We have assessed the perioperative survival probability among trauma patients using the Kaplan–Meier failure estimate. A difference in survival probability between categorical variables was examined using a Log-rank test. Variables that were found to be significant in the Log-rank test were then considered for Cox regression analysis. The semi-parametric Cox proportional hazard regression model was used to identify risk factors associated with perioperative mortality among trauma patients while adjusting for censoring in the data^18^. The adjusted hazard ratio (AHRs) with their corresponding 95% CIs was computed. p-value < 0.05 were considered to be significant. The global Schoenfeld residual test was used to check the proportional hazard assumption (p > 0.05).

We did a sensitivity analysis to check whether including elective surgeries in the analysis affects the association between the risk factors and perioperative mortality among trauma victims. The results of the sensitivity analysis indicate that including or excluding elective surgeries did not affect the association and conclusion (Supplementary Information).

### Ethics approval and consent to participate

This study was approved by the institutional review board (IRB) of the College of Medicine and Health Science, Bahir Dar University (Reference number: 0163/2018), The need for written informed consent was waived for all study subjects by the institutional review board (IRB) of College of Medicine and Health Science, Bahir Dar University and Tibebe Ghion Specialised Hospital. All methods were carried out in accordance with relevant guidelines and regulations.

## Results

### Demographic and pre-hospital characteristics of trauma victims

During the study period, 1106 trauma patients who underwent surgery were recorded. Of these, 37 (3.34%) records were excluded due to incomplete data and 1069 records were included in the final analysis. From all, more than half (53.98%) of trauma victims aged between 18–34, and nearly 84% of trauma patients were males.

### Pre-hospital characteristics

**Figure 1:**
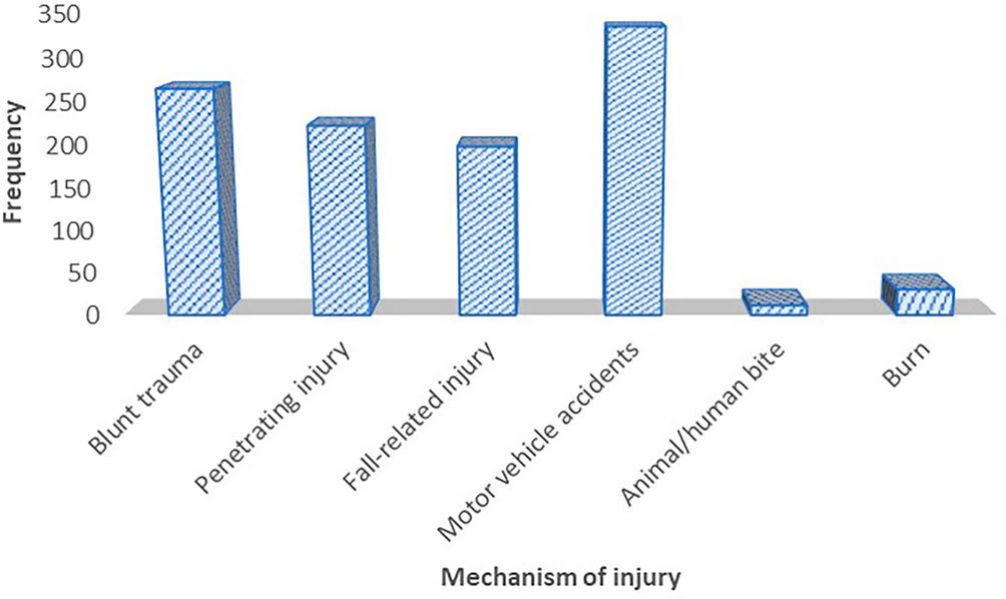
Mechanism of injury among trauma victims.

With regards to time to hospital arrival, about two quarters (48.64%) of trauma victims have arrived after 24 h of traumatic injury, while 415 (38.62%), 100 (9.35%), and 34 (3.18%) cases arrived within 4–24 h, 1–4 h and less than an hour respectively. An ambulance was the most common mode of transportation for trauma patients to the hospital, followed by public transport and private vehicles. Concerning the mechanism of traumatic injury, the top three injuries were motor vehicle accidents, penetration, and blunt trauma (Fig. 1). Most injuries (501) occurred during nighttime, the rest (464) were in the daytime, and only 101 injuries occurred on weekends (Table 1).

**Table 1:** Demographic and pre-hospital characteristics of trauma victims, June 2019–June 2021.

Variable	Category	Frequency	Percentage
Age (in years)	18–34	577	53.98
	34–50	271	25.35
	50–65	140	13.10
	> 65	81	7.58
Sex	Male	895	83.72
	Female	174	16.28
Time to hospital arrival	≤ 1 h	34	3.18
	1–4 h	100	9.35
	4–24 h	415	38.82
	> 24 h	520	48.64
Mode of transport	Ambulance	450	42.82
	Public transport	319	30.15
	Private vehicle	282	26.83
Time frame of trauma	Daytime^a^	464	43.40
	Night^b^	501	47.15
	Weekend^c^	101	9.45

^a^7 AM–5 PM Monday–Friday.

^b^5 PM–7 AM Monday–Friday.

^c^Saturday/Sunday.

### Perioperative characteristics

In this study, most trauma patients (92.24%) exhibit ASA physical status I/II, while 156 study subjects have comorbidity. Three-quarters of trauma victims underwent emergency surgery, and just over half of patients received regional anesthesia. The most commonly performed surgical procedure for trauma patients was orthopedic procedures, followed by neurosurgery and general surgical procedures. Ninety-four (8.79%) trauma victims had a preoperative systolic blood pressure (SBP) below 90 mmHg.

The mean preoperative hemoglobin level was 11.24 (SD 2.56) g/dl. Two hundred fifty-one trauma patients who underwent surgery had more than 500 ml intraoperative blood loss, while 187 had blood transfusions (Table 2).

**Table 2:** Perioperative characteristics of trauma victims, June 2019–June 2021.

Variable	Category	Frequency	Percentage
ASA physical status	I/II	986	92.24
	≥ III	83	7.76
Comorbidity	Yes	156	14.59
	No	913	85.41
Urgency of surgery	Elective	260	24.32
	Emergency	809	75.68
Anesthesia type	General	487	45.56
	Regional	582	54.44
Procedure	Orthopedics	686	64.17
	Neurosurgery	273	25.54
	General surgery	73	6.83
	Others^#^	37	3.46
Hemoglobin (g/dl)^&^	11.24 ± 2.56		
Preoperative SBP (mm Hg)	< 90	94	8.79
	≥ 90	975	81.21
Blood loss (ml)	< 500	818	76.52
	≥ 500	251	23.48
Blood transfusion	Yes	187	17.49
	No	882	82.51

*SBP* systolic blood pressure.

^&^Mean with standard deviation.

^#^Surgeriers including head and neck, cardiothoracic, maxillo-facial, ear, nose, throat (ENT), and gynecological.

### 28-day perioperative mortality among trauma patients

**Figure 2:**
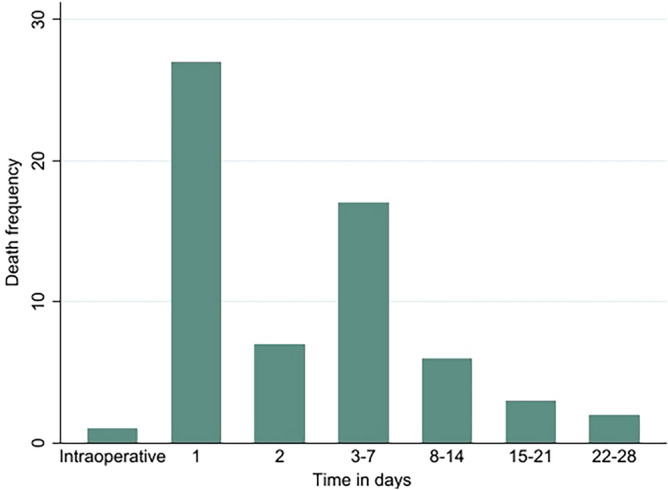
Time distribution of perioperative death among trauma patients, June 2019—June 2021.

**Figure 3:**
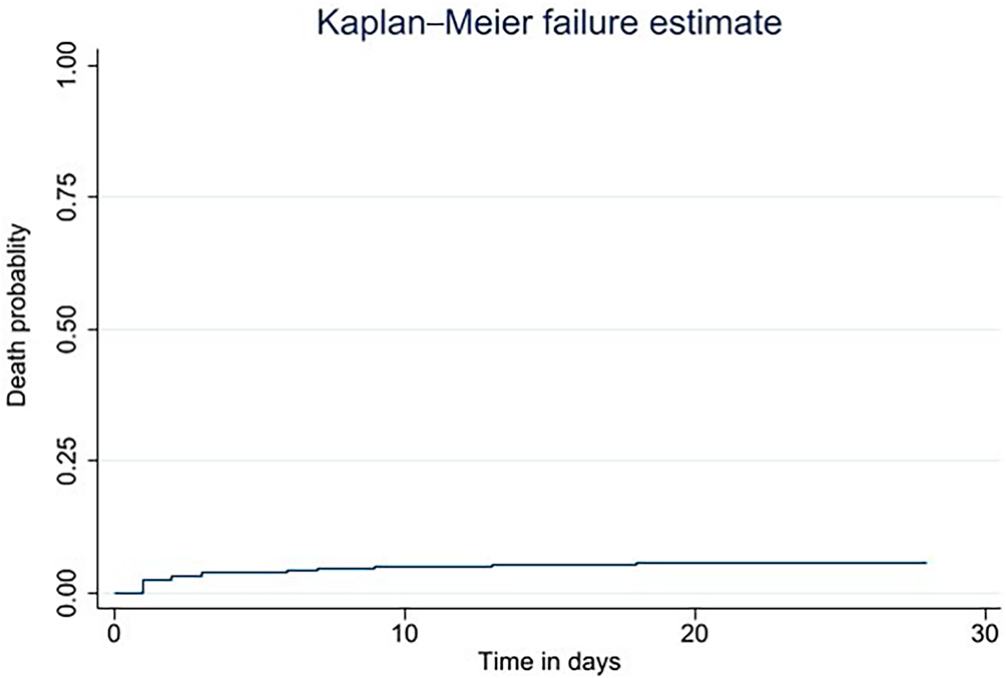
The Kaplan–Meier failure curve of perioperative mortality among trauma patients in Northwest Ethiopia.

One thousand sixty-nine trauma patients were followed until 28 postoperative days with a total of 27,734 person days. During the follow-up, 63 (5.89%) trauma patients died, with an incidence rate of 2.23 (95% CI 1.74 to 2.86) deaths per 1000 person-day observation, and 1006 (94.11%) trauma patients were alive on the 28th postoperative day. Regarding the time of perioperative death, there was one intraoperative death, while 27 deaths occurred on the first postoperative day and seven deaths on the second postoperative day. In addition, there were 17 deaths from the third to the seventh, six deaths from the eighth to the fourteenth, three deaths from the fifteenth to the twenty-first, and two deaths from the twenty-second to the twenty-eighth following the surgery (Fig. 2). Furthermore, the Kaplan-Meir failure curve indicates an increasing trend in the incidence of perioperative deaths over time (Fig. 3).

### Predictors of perioperative mortality among trauma patients

Age ≥ 65 years (AHR = 2.51, 95% CI: 1.04, 6.08), patients sustained blunt trauma (AHR = 3.28, 95% CI: 1.30, 8.29) and MVA (AHR = 2.96, 95% CI: 1.18, 7.43), trauma occurred at night time (AHR = 2.29, 95% CI: 1.15, 4.56), ASA physical status ≥ III (AHR = 3.84, 95% CI: 1.88, 7.82), and blood transfusion (AHR = 2.01, 95% CI: 1.08, 3.74) were identified as a significant risk factor for perioperative mortality among trauma patients (Table 3).

**Table 3:** Predictors of perioperative mortality among trauma patients, June 2019–June 2021.

Variable	Category	Died	Total	CHR (95% CI)	AHR (95% CI)
Age	18 -33	27	577	1	1
	34 – 49	21	271	1.66 (0.94, 2.94)	1.86 (0.80, 3.37)
	50 -64	6	140	0.90 (0.37, 2.19)	0.88 (0.36, 2.17)
	≥ 65	9	81	2.42 (1.14, 5.15)	2.51 (1.04, 6.08)*
Mechanism of injury	Blunt trauma	20	244	2.15 (0.91, 5.09)	3.28 (1.30, 8.29)*
	Penetrating trauma	12	210	1.53 (0.60, 3.90)	1.85 (0.66, 5.17)
	Fall-related	7	191	1	1
	MVA	21	313	1.79 (0.76, 4.23)	2.96 (1.18, 7.43)*
	Others	3	38	2.09 (0.54, 8.08)	3.15 (0.78, 12.79)
Time to hospital arrival	≤ 1 h	3	34	1	1
	1–4 h	3	100	0.32 (0.06, 1.61)	0.60 (0.11, 3.24)
	4–24 h	16	415	0.41 (0.12, 1.43)	0.65 (0.17, 2.44)
	> 24 h	41	520	0.88 (0.27, 2.84)	1.64 (0.46, 5.87)
Mode of transport	Ambulance	29	450	1	1
	Public transport	26	282	1.46 (0.86, 2.49)	2.23 (0.96, 4.02)
	Private vehicle	8	319	0.38 (0.17, 0.84)	0.53 (0.23, 1.19)
Time frame of trauma	Daytime	12	452	1	1
	Night	45	459	3.56 (1.88, 6.74)	2.29 (1.15, 4.56)*
	Weekend	6	95	2.87 (1.07, 7.64)	1.35 (0.47, 3.84)
ASA physical status	I/II	41	945	1	1
	≥ III	22	61	7.35 (4.38, 12.35)	3.84 (1.88, 7.82)**
Comorbidity	Yes	20	156	2.76 (1.62, 4.70)	1.24 (0.63, 2.43)
	No	43	913	1	1
Urgency of surgery	Elective	5	260	1	1
	Emergency	58	809	3.79 (1.52, 9.46)	2.23 (0.82, 6.05)
Anesthesia type	General	39	487	1.96 (1.18. 3.27)	0.96 (0.52. 1.79)
	Regional	24	582	1	1
Hemoglobin (g/dl)		11.04 ± 2.76	11.24 ± 2.56	1.00 (0.90, 1.10)	
Preoperative SBP (mm Hg)	< 90	11	94	2.45 (1.27, 4.70)	2.07 (0.91, 4.23)
	≥ 90	52	975	1	1
Blood loss (ml)	< 500	38	818	1	1
	≥ 500	25	251	2.22 (1.34, 3.67)	1.27 (0.68, 2.34)
Blood transfusion	Yes	25	162	3.22 (1.94, 5.34)	2.01 (1.08, 3.74)*
	No	38	844	1	1

*AHR* adjusted hazard ratio, *CHR* crude hazard ratio.

*p < 0.05, **p < 0.001.

## Discussion

The main objective of this study was to report perioperative mortality rates among adult trauma victims in a tertiary hospital in Ethiopia. With an incidence rate of 2.23 (95% CI 1.74 to 2.86) deaths per 1000 person-day observation, the current study found that the 28-day perioperative mortality rate among trauma patients at Tibebe Ghion Specialized Hospital was 5.89%. This is in line with a study done in Nigeria^19^. The mortality rate in this study was lower than those of high- and middle-income countries^8,20^. The possible explanation for this discrepancy might be a difference in sample size and study population. Our study used a small sample size and included elective surgeries, which might underestimate the perioperative mortality rate. In addition, in our setting, where trauma referral systems are poor, most critically traumatized patients could not reach the hospital alive; therefore, most deaths might occur before hospital arrival, affecting the frequency of perioperative deaths.

In this study, a high proportion of trauma victims arrived at the hospital after 24 h of injury time, indicating a poor trauma care response system, as seen in most LICs. The flawed trauma care response system has a detrimental impact on the outcome of trauma victims^21^. A previous study in Northwest Ethiopia noted that establishing a trauma care response system is crucial and should be established at any cost^22^. Establishing a trauma care response system incorporating trauma centers and implementing quality improvement programs on trauma care are well-known strategies to improve the outcomes of trauma victims^16,21,23^.

Age ≥ 65 years, patients sustained blunt trauma and MVA, trauma occurred at night time, ASA physical status ≥ III, and blood transfusion were identified as significant risk factors for perioperative mortality among trauma patients. As supported by several studies^24–26^, older trauma patients were shown to have an elevated perioperative mortality risk following traumatic injuries. The reason might be that elderlies, due to aging, will have pre-existing medical conditions, diminished physiologic reserve, and the inability to compensate for severe injuries, which increase the risk of perioperative death^27^. Additionally, elderly patients are at a greater risk of experiencing post-traumatic complications, which is linked to a shift toward delayed mortality^28^.

The finding of this study revealed that trauma victims who sustained traumatic injuries during nighttime have an increased hazard of death compared to those victims who suffered injuries during daytime and weekends. This finding agrees with a study in Japan^29^; the explanation could be that injuries sustained at night are likely to be severe, increasing the risk of death^30^. Compared to other mechanisms of injury, patients who sustained blunt trauma and MVA had a more than threefold increase in mortality hazard. This finding is consistent with a study by Kleber et al.^31^, which reports a high incidence of death among patients who sustained blunt trauma compared with penetrating, and a study by Loberg et al.^32^, which reports motor vehicle trauma as a risk factor for in-hospital mortality among trauma patients. Clinical diagnosis of victims of blunt trauma is difficult, such as concealed hemorrhage and other site injuries, leading to a late or missed diagnosis. Victims who sustain MVA are highly likely to suffer from traumatic brain injury and spinal cord injury, which negatively affect the chance of survival^33^.

ASA physical status score is an independent predictor of mortality among trauma patients^34,35^. In our study, trauma patient with a higher ASA physical status score has an increased hazard of perioperative mortality compared to those with a lower score. The best explanation for this might be that higher ASA physical status scores indicate severe disease conditions with an increased risk of death. In our study, trauma victims who received blood transfusion intraoperatively have a higher risk of perioperative death than their counterparts. The possible explanation might be the occurrence of transfusion-related complications such as allergic reactions, fever, and hemolytic reactions, which increase the likelihood of death perioperatively. In addition, the risk of post-injury infection and multi-organ failure is higher among patients who received blood transfusion, which might increase the risk of perioperative death^36^.

The findings of our study provide valuable baseline information on perioperative outcomes among trauma victims in Northwest Ethiopia. Furthermore, our results can assist clinicians handling trauma victims during the perioperative period in implementing evidence-based practice and prognosis-tailored treatment. The study highlights the importance of addressing the unique needs of trauma patients who are at a higher risk of perioperative death.

### Limitation

The main limitation of this study was that we did not include trauma scoring systems such as the Glasgow Come Scale (GCS), Revised Trauma Score (RTS), and Injury Severity Score (ISS), which are well-known predictors for trauma victims' outcomes. In addition, our study had a diverse surgical population, which might affect the identification of perioperative mortality predictors. Furthermore, this study was conducted in a single center, which can affect the generalizability of the study.

## Conclusion

In this trauma cohort, it was demonstrated that perioperative mortality is a healthcare burden. Most trauma victims come after 24 h of sustained injuries, which shows a poor pre-hospital emergency service. Risk factors for perioperative mortality among trauma patients were old age, patients sustaining blunt trauma and MVA, trauma occurring at night, higher ASA physical status, and blood transfusion.

Based on the results of this study, trauma care services need improvement in pre-hospital and perioperative care. Trauma care providers should implement specially targeted interventions for victims with identified risk factors.

### Supplementary Information

Supplementary Tables.

## Data Availability

The data generated during and analyzed during this study are available from the corresponding request upon a reasonable request.

## Abbreviations

AHR: Adjusted hazard ratio
ASA: American Society of Anesthesiologist
CHR: Crude hazard ratio
LIC: Low income countries
LMIC: Low-and-middle-income countries
MVA: Motor vehicle accident

## References

[1] *Injuries and Violence: World Health Organization* (*WHO*). https://www.who.int/news-room/fact-sheets/detail/injuries-and-violence. Accessed 19 Mar 2021.

[2] Mock C, Joshipura M, Arreola-Risa C, Quansah R (2012). An estimate of the number of lives that could be saved through improvements in trauma care globally. World J. Surg..

[3] Haagsma JA, Graetz N, Bolliger I, Naghavi M, Higashi H, Mullany EC (2016). The global burden of injury: Incidence, mortality, disability-adjusted life years and time trends from the Global Burden of Disease study 2013. Inj. Prev..

[4] Shanthakumar D, Payne A, Leitch T, Alfa-Wali M (2021). Trauma care in low- and middle-income countries. Surg. J. (N. Y.).

[5] Negoi I, Paun S, Hostiuc S, Stoica B, Tanase I, Negoi RI (2015). Mortality after acute trauma: Progressive decreasing rather than a trimodal distribution. J. Acute Dis..

[6] Gunst, M., Ghaemmaghami, V., Gruszecki, A., Urban, J., Frankel, H. & Shafi, S. (eds.) Changing epidemiology of trauma deaths leads to a bimodal distribution. In *Baylor University Medical Center Proceedings* (Taylor & Francis, 2010). 10.1080/08998280.2010.11928649 PMC2943446 20944754

[7] Denu ZA, Yassin MO, Azale T, Biks GA, Gelaye KA (2021). Do deaths from road traffic injuries follow a classical trimodal pattern in North West Ethiopia? A hospital-based prospective cohort study. BMJ Open.

[8] Mansourati M, Kumar V, Khajanchi M, Saha ML, Dharap S, Seger R (2018). Mortality following surgery for trauma in an Indian trauma cohort. Br. J. Surg..

[9] Ekeke ON, Okonta KE (2017). Trauma: A major cause of death among surgical inpatients of a Nigerian tertiary hospital. Pan Afr. Med. J..

[10] Mehmood A, Rowther AA, Kobusingye O, Ssenyonjo H, Zia N, Hyder AA (2021). Delays in emergency department intervention for patients with traumatic brain injury in Uganda. Trauma Surg. Acute Care Open.

[11] Braz LG, Carlucci MT, Braz JRC, Módolo NS, do Nascimento Jr P, Braz MG (2020). Perioperative cardiac arrest and mortality in trauma patients: A systematic review of observational studies. J. Clin. Anesth..

[12] Endalamaw A, Birhanu Y, Alebel A, Demsie A, Habtewold TD (2019). The burden of road traffic injury among trauma patients in Ethiopia: A systematic review and meta-analysis. Afr. J. Emerg. Med..

[13] Ananya TG, Sultan M, Zemede B, Zewdie A (2021). Pre-hospital care to trauma patients in Addis Ababa, Ethiopia: Hospital-based cross-sectional Study. Ethiop. J. Health Sci..

[14] Meshesha BR, Sibhatu MK, Beshir HM, Zewude WC, Taye DB, Getachew EM (2022). Access to surgical care in Ethiopia: A cross-sectional retrospective data review. BMC Health Serv. Res..

[15] Dumovich, J.S.P. Physiology, trauma: In *StatPearls* [*Internet*]. https://www.ncbi.nlm.nih.gov/books/NBK538478/. Accessed 19 Sep 2023 (StatPearls Publishing, 2022)

[16] Bayissa BB, Alemu S (2021). Pattern of trauma admission and outcome among patients presented to Jimma University Specialized Hospital, south-western Ethiopia. Trauma Surg. Acute Care Open.

[17] Sileshi B, Newton MW, Kiptanui J, Shotwell MS, Wanderer JP, Mungai M (2017). Monitoring anesthesia care delivery and perioperative mortality in Kenya utilizing a provider-driven novel data collection tool. Anesthesiology..

[18] Deo SV, Deo V, Sundaram V (2021). Survival analysis—Part 2: Cox proportional hazards model. Indian J. Thorac. Cardiovasc. Surg..

[19] Ekeke ON, Okonta KE (2017). Trauma: A major cause of death among surgical inpatients of a Nigerian tertiary hospital. Pan Afr. Med. J..

[20] Braz LG, Carlucci MTO, Braz JRC, Módolo NSP, do Nascimento P, Braz MG (2020). Perioperative cardiac arrest and mortality in trauma patients: A systematic review of observational studies. J. Clin. Anesth..

[21] Reynolds TA, Stewart B, Drewett I, Salerno S, Sawe HR, Toroyan T (2017). The impact of trauma care systems in low-and middle-income countries. Annu. Rev. Public Health.

[22] Denu ZA, Osman MY, Bisetegn TA, Biks GA, Gelaye KA (2022). Barriers and opportunities of establishing an integrated pre-hospital emergency response system in North West Ethiopia: A qualitative study. Injury Prevent..

[23] Choi J, Carlos G, Nassar AK, Knowlton LM, Spain DA (2021). The impact of trauma systems on patient outcomes. Curr. Probl. Surg..

[24] Li LF, Lui WM, Wong HH, Yuen WK, Leung GK (2017). Outcome after operative intervention for traumatic brain injuries in the elderly. Asian J. Neurosurg..

[25] Bedada AG, Tarpley MJ, Tarpley JL (2021). The characteristics and outcomes of trauma admissions to an adult general surgery ward in a tertiary teaching hospital. Afr. J. Emerg. Med..

[26] Busse J, Bhandari M, Devereaux PJ (2004). The impact of time of admission on major complications and mortality in patients undergoing emergency trauma surgery. Acta Orthopaed. Scand..

[27] Victorino GP, Chong TJ, Pal JD (2003). Trauma in the elderly patient. Arch. Surg..

[28] Hildebrand F, Pape H-C, Horst K, Andruszkow H, Kobbe P, Simon T-P (2016). Impact of age on the clinical outcomes of major trauma. Eur. J. Trauma Emerg. Surg..

[29] Hirose T, Kitamura T, Katayama Y, Sado J, Kiguchi T, Matsuyama T (2020). Impact of nighttime and weekends on outcomes of emergency trauma patients: A nationwide observational study in Japan. Medicine..

[30] Riyapan S, Chantanakomes J, Somboonkul B, Shin SD, Chiang W-C (2022). Effect of nighttime on prehospital care and outcomes of road traffic injuries in Asia: A cross-sectional study of data from the Pan-Asian Trauma Outcomes Study (PATOS). Prehosp. Emerg. Care.

[31] Kleber C, Giesecke MT, Tsokos M, Haas NP, Schaser KD, Stefan P (2012). Overall distribution of trauma-related deaths in Berlin 2010: Advancement or stagnation of German trauma management?. World J. Surg..

[32] Loberg JA, Hayward RD, Fessler M, Edhayan E (2018). Associations of race, mechanism of injury, and neighborhood poverty with in-hospital mortality from trauma: a population-based study in the Detroit metropolitan area. Medicine.

[33] Zheng X-Y, Yi Q, Xu X-J, Meng R-L, Ma S-L, Tang S-L (2021). Trends and external causes of traumatic brain injury and spinal cord injury mortality in south China, 2014–2018: An ecological study. BMC Public Health.

[34] Skaga NO, Eken T, Søvik S, Jones JM, Steen PA (2007). Pre-injury ASA physical status classification is an independent predictor of mortality after trauma. J. Trauma Acute Care Surg..

[35] Kuza CM, Matsushima K, Mack WJ, Pham C, Hourany T, Lee J (2019). The role of the American Society of anesthesiologists physical status classification in predicting trauma mortality and outcomes. Am. J. Surg..

[36] Charles A, Shaikh A, Walters M, Huehl S, Pomerantz R (2007). Blood transfusion is an independent predictor of mortality after blunt trauma. Am. Surg..

