# Supplementary material for: Perioperative mortality among trauma patients in Northwest Ethiopia: a prospective cohort study
Source: Sci Rep. 2023 Dec 21;13:22859. doi: 10.1038/s41598-023-50101-8 (PMC10739862; doi:10.1038/s41598-023-50101-8)
Supplement: Supplementary file 1 — Supplementary Tables. [file 41598_2023_50101_MOESM1_ESM.docx]

Table: Multivariable Cpx regression after excluding elective surgeries

| _t | Category | Haz. ratio | Std. err. | z | P>z | [95% conf. interval] |
| --- | --- | --- | --- | --- | --- | --- |
| Age | 18-33 | 1 |  |  |  |  |
|  | 34-49 | 1.830479 | .5539229 | 2.00 | 0.08 | .911537 3.312437 |
|  | 50-64 | .8947061 | .4105728 | -0.24 | 0.808 | .363975 2.199325 |
|  | >65 | 2.564203 | 1.162547 | 2.08 | 0.038 | 1.05448 6.235431 |
| Time to hospital arrival | ≤ 1 hour | 1 |  |  |  |  |
|  | 1- 4 hour | .6236958 | .5330539 | -0.55 | 0.581 | .1168091 3.330189 |
|  | 4 -24 hour | .6926813 | .4646566 | -0.55 | 0.584 | .1860116 2.579448 |
|  | > 24 hours | 1.663958 | 1.077146 | 0.79 | 0.432 | .4678707 5.917783 |
| Mode of transport | Ambulance | 1 |  |  |  |  |
|  | Private vehicle | 2.134433 | .6386933 | 2.53 | 0.091 | .887339 3.836987 |
|  | Public transportation | .5290458 | .2209203 | -1.52 | 0.127 | .2333707 1.199334 |
| Time frame of trauma | Daytime | 1 |  |  |  |  |
|  | Night | 2.808176 | .9497834 | 3.05 | 0.002 | 1.447206 5.449019 |
|  | Weekend | 1.662839 | .8673844 | 0.97 | 0.330 | .59819 4.622334 |
| ASA physical status | I/II | 1 |  |  |  |  |
|  | ≥III | 3.933323 | 1.428729 | 3.77 | 0.000 | 1.930063 8.015815 |
| Comorbidity | Yes | 1.209886 | .4138095 | 0.56 | 0.577 | .6188902 2.365241 |
|  | No | 1 |  |  |  |  |
| Anesthesia type | General | 1.052503 | .3314186 | 0.16 | 0.871 | .5677956 1.95099 |
|  | Regional | 1 |  |  |  |  |
| Mechanism of injury | Blunt trauma | 3.405825 | 1.619457 | 2.58 | 0.010 | 1.341155 8.648993 |
|  | Penetrating injury | 1.948822 | 1.028547 | 1.26 | 0.206 | .6926698 5.483 |
|  | Fall related | 1 |  |  |  |  |
|  | MVA | 3.033156 | 1.436358 | 2.34 | 0.019 | 1.198964 7.673318 |
|  | Other | 3.261699 | 2.334028 | 1.65 | 0.099 | .8022975 13.26027 |
| Systolic BP | <90 | 2.047284 | .7470889 | 1.96 | 0.09 | .991291 4.18597 |
|  | >90 | 1 |  |  |  |  |
| Blood loss | <500 | 1 |  |  |  |  |
|  | ≥500 | 1.270361 | .3956673 | 0.77 | 0.442 | .6899406 2.339068 |
| Blood transfusion | Yes | 2.037715 | .6454191 | 2.25 | 0.025 | 1.095309 3.790968 |
|  | No | 1 |  |  |  |  |

Table: Global and scaled Schonefeld residual assessing proportional hazard assumption

| Variable | rho | chi2 | df | Prob>chi2 |
| --- | --- | --- | --- | --- |
| Age | 0.21603 | 3.00 | 1 | 0.0831 |
| Time to hospital arrival | -0.18846 | 3.26 | 1 | 0.0709 |
| Mode of transport | -0.16311 | 1.11 | 1 | 0.2916 |
| Time frame of trauma | -0.25636 | 2.59 | 1 | 0.1078 |
| ASA physical status | 0.17464 | 1.98 | 1 | 0.1589 |
| Urgency of surgery | 0.18437 | 1.82 | 1 | 0.1778 |
| Comorbidity | -0.12700 | 0.78 | 1 | 0.3764 |
| Anesthesia type | 0.17588 | 1.69 | 1 | 0.1937 |
| Mechanism of injury | -0.10372 | 0.93 | 1 | 0.3337 |
| Preoperative SBP | -0.10143 | 0.69 | 1 | 0.4073 |
| Blood loss | -0.05208 | 0.18 | 1 | 0.6690 |
| Blood transfusion | 0.12239 | 1.02 | 1 | 0.3118 |
| Hemoglobin | -0.11136 | 0.97 | 1 | 0.3257 |
| Global test |  | 23.14 | 13 | 0.1400 |
|  |  |  |  |  |

Table: Log-rank for the predictors of perioperative mortality among trauma patients

| **Variable** | **df** | **chi2** | **Pr>chi2** |
| --- | --- | --- | --- |
| Age | 3 | 7.84 | 0.0495 |
| Sex | 1 | 0.02 | 0.9009 |
| Time to hospital arrival | 3 | 9.17 | 0.0271 |
| Mode of transport | 2 | 12.38 | 0.0021 |
| Time frame of trauma | 2 | 17.65 | 0.0001 |
| Mechanism of injury | 4 | 16.58 | <0.0001 |
| ASA physical status | 1 | 79.50 | <0.0001 |
| Comorbidity | 1 | 15.59 | 0.0001 |
| Urgency of surgery | 1 | 9.61 | 0.0019 |
| Anesthesia type | 1 | 7.20 | 0.0073 |
| Procedure | 3 | 3.52 | 0.4750 |
| Preoperative SBP | 1 | 7.87 | 0.0050 |
| Blood loss | 1 | 10.24 | 0.0014 |
| Blood transfusion | 1 | 23.47 | <0.0001 |
